# Intraguild Predation Behaviour of Ladybirds in Semi-Field Experiments Explains Invasion Success of *Harmonia axyridis*

**DOI:** 10.1371/journal.pone.0040681

**Published:** 2012-07-16

**Authors:** C. Lidwien Raak-van den Berg, Hendrika J. De Lange, Joop C. Van Lenteren

**Affiliations:** 1 Laboratory of Entomology, Wageningen University, Wageningen, The Netherlands; 2 Alterra, Wageningen UR, Wageningen, The Netherlands; University of Utah, United States of America

## Abstract

*Harmonia axyridis* has been introduced as a biological control agent in Europe and the USA. Since its introduction, it has established and spread, and it is now regarded as an invasive alien species. It has been suggested that intraguild predation is especially important for the invasion success of *H. axyridis*. The aim of this study was to compare the intraguild predation behaviour of three ladybird species (*Coccinella septempunctata, Adalia bipunctata,* and *H. axyridis*). Predation behaviour was investigated in semi-field experiments on small lime trees (*Tilia platyphyllos*). Two fourth-instar larvae placed on a tree rarely made contact during 3-hour observations. When placed together on a single leaf in 23%–43% of the observations at least one contact was made. Of those contacts 0%–27% resulted in an attack. *Harmonia axyridis* attacked mostly heterospecifics, while *A. bipunctata* and *C. septempunctata* attacked heterospecifics as often as conspecifics. In comparison with *A. bipunctata* and *C. septempunctata*, *H. axyridis* was the most successful intraguild predator as it won 86% and 44% of heterospecific battles against *A. bipunctata* and *C. septempunctata* respectively, whilst *A. bipunctata* won none of the heterospecific battles and *C. septempunctata* won only the heterospecific battles against *A. bipunctata. Coccinella septempunctata* dropped from a leaf earlier and more often than the other two species but was in some cases able to return to the tree, especially under cloudy conditions. The frequency with which a species dropped did not depend on the species the larva was paired with. The results of these semi-field experiments confirm that *H. axyridis* is a strong intraguild predator as a consequence of its aggressiveness and good defence against predation from heterospecific species. The fact that *H. axyridis* is such a strong intraguild predator helps to explain its successful establishment as invasive alien species in Europe and the USA.

## Introduction

Since its introduction as a biological control agent, *Harmonia axyridis* (Pallas) (Coleoptera, Coccinellidae) has established and spread. It is now regarded as an invasive alien species in both Europe and the USA. The ladybird is no longer commercially available in most of Europe [1] as it has a negative impact on non-target insect species, fruit production, and human health [2], [3], [4]. The invasiveness of *H. axyridis* has also raised concerns about the fate of populations of native coccinellids [5], [6], [7] and the impact of this species on the intricate multitrophic aphidophagous food web [8].

*Harmonia axyridis* is cannibalistic and successfully preys upon larvae and eggs of other aphid predators (intraguild predation). It has been suggested that intraguild predation (IGP) is one of the reasons for the success of *H. axyridis* as an invasive species [8], [9], [10]. IGP is defined as the killing and eating of species that use similar, often limited, resources and is a well-known phenomenon across a wide range of taxa, such as fish, invertebrates, and mammals (e.g. [11]). Aphidophagous guilds are systems in which IGP is one of the main forces influencing population structure and dynamics [8], [12]. IGP and cannibalism, are suspected to have developed as a result of scarcity or absence of the main prey [13], [14], [15]. In general, the presence of extraguild prey can reduce the occurrence and intensity of IGP (e.g. [16]). Oviposition and larval development of *Coccinella septempunctata* L. (Coleoptera: Coccinellidae) and *Adalia bipunctata* L. (Coleoptera: Coccinellidae) are synchronised with the aphid population peak in northwestern Europe. *Harmonia axyridis*, however, arrives later and has to complete its development when aphid densities are low [17], [18], [19].

This late arrival of *H. axyridis* is thought to have resulted in a higher dependence on cannibalism and IGP, which probably explains its aggressive nature and successful defence strategies [17], [18], [19]. Indeed, there is strong evidence from laboratory experiments that, within the aphidophagous guild, *H. axyridis* is a strong, if not the strongest intraguild predator (e.g. [8], [9], [10], [12]). Its higher mobility, increased levels of aggressiveness [5], [16], [20], and larger size [10], [21] seem to be important factors in the success of *H. axyridis* as an intraguild predator.

Coccinellid larvae can defend themselves against IGP by using a range of behavioural, physiological and morphological strategies, which include; running away or dropping [22], release of toxic alkaloids [23], and the presence of features such as dorsal spines [9], [10], [24]. Most evidence for IGP behaviour is based on laboratory experiments [12], [25], [26], but the results are difficult to extrapolate to field conditions due to the increased complexity and variation within and between wild habitats. Structured habitats provide refuge to intraguild prey [27], so intraguild prey suffers less from predation [28], and the availability of different host plants may also influence IGP pressure [29]. Further, alternative food sources, daily and yearly differences in activity cycles, and the possibilities to avoid confrontation and to escape will reduce IGP events [25], [30]. As the understanding of all IGP-relations in aphidophagous guilds is hampered by the bias of laboratory experiments, there is a great need for experimental studies under field conditions or semi-field conditions which more closely approximate field conditions than a traditional laboratory setup.

The aim of this study was to compare the IGP-behaviour of three ladybird species under semi-field conditions. Experiments were performed with two native European species (*C. septempunctata* and *A. bipunctata*) and the invasive alien species *H. axyridis*. The three species we studied use different defence mechanisms against IGP. *Coccinella septempunctata* is defended by size (against *A. bipunctata* but not against *H. axyridis* [10]), by dropping behaviour [22] and, to a certain extent, by defensive chemicals [13], [31]. *Adalia bipunctata* uses chemical defences, which protect it against *C. septempunctata* (e.g. [13], [31]), but not completely against *H. axyridis* [22], [31]. *Harmonia axyridis* defends itself by size [10], by chemical deterrence (reviewed by [32]), and by morphological structure (spines) [24]. Small lime trees (*Tilia platyphyllos* Scop. (Malvales, Malvaceae)) were used as natural host plants for the ladybirds. The following research questions were addressed: (1) how often do two larvae of different or the same species come into contact? (2) what happens when they make contact? (3) which species generally wins the interaction, and (4) can the species be ranked on the basis of the outcome of the interaction? These questions were successfully investigated in two different experimental set-ups: on individual lime tree leaves, where escape responses may affect the interactions, and on whole lime trees, where escape responses along with encounter rates may affect the interactions.

## Materials and Methods

### Ethics Statement

Experiments were conducted according to the Dutch national regulations: No additional admission is needed for invertebrates.

### Insects

*Harmonia axyridis* adults were collected from hibernation sites in Kootwijk on 13 November 2009 (location N 51 59 32, E 5 39 43) and Houten on 27 November 2009 (location N 52 1 39, E 5 9 38), the Netherlands. *Adalia bipunctata* adults were also collected at those sites and at various other locations. All *A. bipunctata* individuals were found within aggregations of *H. axyridis*. All collected beetles were kept in a climate cabinet at 5°C ±1, 0∶24 L:D to continue overwintering and transferred to a climate chamber at 24°C ±1, 16∶8 L:D, 55% ±5 RH in May 2010. In May and early June 2010, adults of *C. septempunctata* and *A. bipunctata* were collected in Wageningen, the Netherlands (location N 52 10 39, E 5 45 39) and transferred to the same climate chamber at 24°C.

Before the start of the experiments, the beetles were sexed and paired. Eighteen pairs of *H. axyridis*, twenty pairs of *C. septempunctata*, and ten pairs of *A. bipunctata* were formed. Two *C. septempunctata* females and eight *A. bipunctata* females were laying fertile eggs and were also used. When one adult of a pair died, the surviving adult was paired with a new individual. Each pair or individual female was kept in a Petri dish (Ø 9 cm) lined with filter paper and a folded strip of filter paper as substrate for oviposition. All beetles were given honey water and pollen *ad libitum*. In addition, *C. septempunctata* was daily fed pea aphids (*Acyrthosiphon pisum* Harris (Hemiptera: Aphididae) reared on *Vicia faba* L. (Fabales, Fabaceae)) *ad libitum*, and *H. axyridis* and *A. bipunctata* were fed dead, irradiated eggs of *Ephestia kuehniella* Zeller (Lepidoptera: Pyralidae) *ad libitum* and pea aphids three times a week. Eggs and aphids were provided by Koppert Biological Systems, Berkel en Roderijs, the Netherlands.

Egg batches were collected daily. Three to six first-instar larvae from each batch were placed individually in Petri dishes and fed with *E. kuehniella* (*H. axyridis* and *A. bipunctata*) or pea aphids (*C. septempunctata*) *ad libitum*. *Coccinella septempunctata* larvae were also given water by means of moistened cotton wool. Third-instar *H. axyridis* and *A. bipunctata* larvae were fed pea aphids once. Within 24 hours after moult into the fourth-instar, all larvae were starved for 24 hours, with access to water by means of moistened cotton wool.

Starved fourth-instar individuals were used in the experiments in six combinations: 1. *H. axyridis* & *H. axyridis*, 2. *C. septempunctata* & *C. septempunctata*, 3. *A. bipunctata* & *A. bipunctata*, 4. *H. axyridis* & *C. septempunctata,* 5. *H. axyridis* & *A. bipunctata*, and 6. *C. septempunctata* & *A. bipunctata*. For each observation larvae from different parents were combined in order to maximise variation. The larvae were marked with Uni Posca pigment markers (a water based-acrylic paint) to allow for individual recognition. A pilot test showed that this way of marking allows larvae to move and to develop normally into the next instar. Experiments were conducted in a large cage (4 m×12 m×3 m) to keep the trees free from aphid infestation. To avoid disturbance by rain, the roof of the cage was covered with a plastic sheet. Temperature was recorded using a Hobo ProV2 temperature logger (MicroDaq.com, Ltd., Contoocook NH, USA). All experiments were conducted in the period from 4 June to 9 July 2010.

### Leaf Experiment

The behaviour of two larvae encountering each other on a leaf was observed. Compared to studies performed in Petri dishes, the size of the leaf formed a comparable area for interactions to take place but the larvae had the opportunity to escape, which may be an important outcome of interactions in field conditions. The experiment was conducted on individual leaves of the trees that had been used in the tree experiment (see next section); the leaves had an average surface area of 140 cm^2^.

Larvae may react to (fresh) larval tracks; however, there is no clear evidence for the persistence of these tracks [33], [34]. Moreover, Moser et al. [35] postulate that larval tracks play only a minor role in foraging behaviour of *H. axyridis*. For adult female ladybirds the persistence of the oviposition-deterrent effect of larval tracks has been shown to be 5 to 10 days [36], [37]. Therefore, individual leaves were used at least 5 days after the tree experiment had been conducted, to allow the larval tracks to diminish.

**Figure 1:**
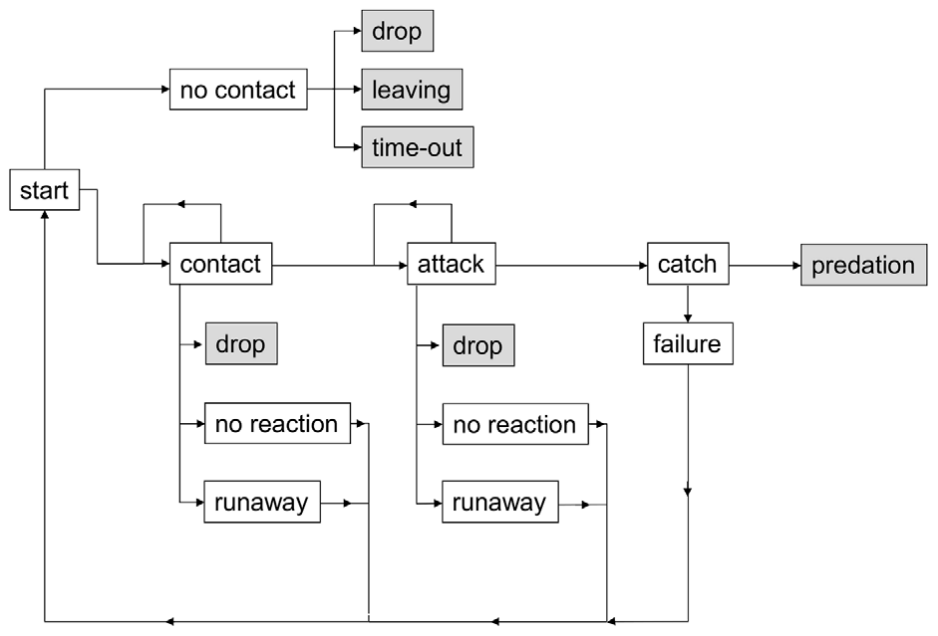
Behavioural sequence of coccinellid larval encounters. Grey shaded behaviour indicates end of the observation (adapted from Yasuda et al., 2001 [20]).

For each observation, two larvae were gently placed on the surface of a horizontally positioned leaf that had not been used before in the leaf experiment. After the second larva was placed on the leaf, the behaviour of the two individuals was recorded continuously with The Observer XT 10.0. An observation was ended when: 1) one larva left the leaf by dropping from the leaf; 2) one larva walked off the leaf onto the branch (henceforward referred to as “leaving”); 3) one larva attacked, caught, and preyed upon the other larva; or 4) after 1000 seconds, when none of the other three options occurred (henceforward referred to as “time-out”). We used a behavioural sequence adapted from Yasuda et al. [20] (figure 1). A distinction was made between actor ( = the acting individual) and reactor ( = the responding individual). A counter contact or counter attack resulted in a change in actor and reactor. Behaviour after contact was divided into aggressive responses by the actor (attack, catch, predation), and non-aggressive responses by the reactor (no reaction, runaway, drop). Runaway and drop were considered escape responses. During some observations multiple contacts were made; these were treated as independent events in the statistical analysis if the time between two consecutive contacts was more than ten seconds. Each day that the experiment was conducted, at least one replicate of each treatment (species combination) was tested. Replicates and treatments were executed randomly. The number of replicates per treatment and the total number of replicates depended on the number of larva available and on logistic constraints.

### Tree Experiment

In this experiment the spatial scale was increased even more than in the leaf experiment, as the larvae were observed on whole trees: interactions were probably not only affected by escape rates but also by encounter rates. Two-year-old lime trees (*T. platyphyllos*) in 10L containers were used. Each tree was pruned to one 50-cm stem and watered daily. On a table (85 cm×560 cm) six consecutive arenas (85 cm×85 cm) were constructed from stiff, black plastic boarding (50 cm high), allowing us to test the six species combinations simultaneously. The arenas were filled with a 30 cm layer of white sand. Tanglefoot at the inner side of the plastic boarding prevented the larvae from escaping. On the day of the experiment one container with one tree (with on average eight branches and 53 leaves) was placed in each arena. The container was buried in sand, creating a flat surface stretching from the boarding to the tree stem. Two larvae were placed on the upper sides of two leaves at two opposite sides of the tree, on average 50 cm from each other. The position of the larvae on the tree and their behaviour were recorded every five minutes for three hours. Table 1 provides the definitions of the behavioural categories. The experiment was repeated 15 times, resulting in fifteen 3-hour observations of each of the six different larval combinations, summing up to a total of 90 observations. Larval combinations were rotated between the different arenas so that spatial differences could be accounted for. All observations started approximately at 11∶30 am and were recorded with The Observer XT 10.0 (Noldus Information Technology B.V., Wageningen, The Netherlands). After 24 hours, the fate of the larvae (alive or dead) and their position was recorded.

**Table 1:** Description of larval coccinellid behaviour.

Behaviour	Actor or reactor	Description
contact	actor	larvae touch each other with any body part
attack	actor	larva attacks other larva
catch	actor	larva catches other larva
predation	actor	larva preys upon other larva
counter contact	reactor	larva reacts to contact with new contact
runaway	reactor	larva runs away after contact, faster than normal walking speed
no reaction	reactor	larva does not change behaviour after contact or attack
counter attack	reactor	larva reacts to attack with new attack
failure	reactor	larva struggles itself free after being caught
drop	reactor/end experiment	one of the larvae drops from leaf
leaving	end experiment	one of the larvae walks over petiole onto branch
time-out	end experiment	1000 seconds have passed without the experiment ending by drop, leaving, or predation

The actor is the acting individual; the reactor is the responding individual.

### Data Analysis

**Figure 2:**
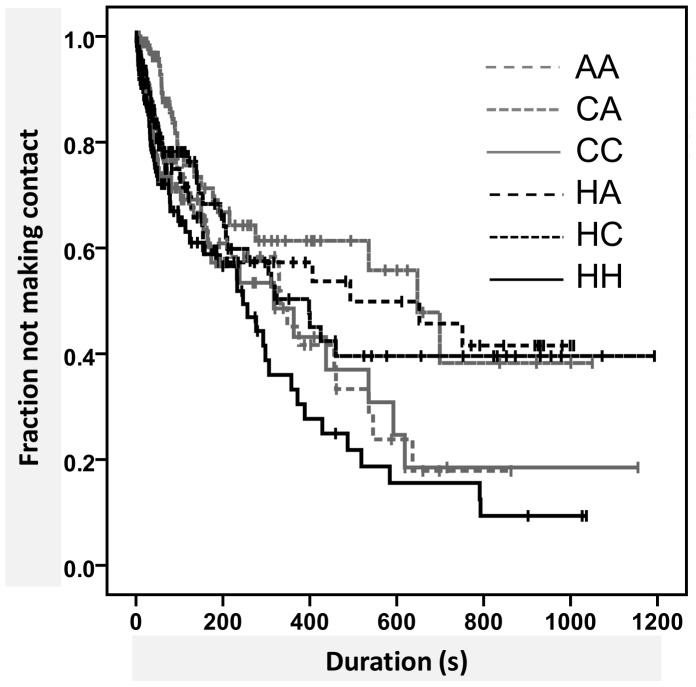
Effect of species combination on time until first contact. Survival curves of difference in time until contact between fourth-instar larvae per species combination during 1000-second observations. Censored observations are marked with ‘+’. Abbreviations: H = *H. axyridis*, C = *C. septempunctata*, and A = *A. bipunctata*. Time until contact significantly differs between combinations when all contacts are pooled together (Log-Rank test, pooled pairwise comparisons, P = 0.008).

In a large number of the observations during the leaf experiment, the larvae did not make contact with one another, resulting in a many censored data. Survival analysis is then an appropriate technique to analyse the time from the start of an observation until the first contact, since this approach incorporates the time until a certain event occurs and includes censored data [38]. An event is defined as an *a priori*-defined incident that happens to an individual. In this study an event was the contact between larvae, and censored data were situations where the experiment was ended before the event had occurred (e.g. time-out, leaving without contact, or drop without contact). Data were plotted with Kaplan-Meier’s product limit estimator. The Log-rank test was used to test whether covariates had a significant effect on the time until contact for the different species (combinations). As the survivor curves of the species do not necessarily have a similar form (see figure 2) these differences cannot be analysed with Cox’s proportional hazards model [38]. The effect of temperature on the time until contact was analysed with Cox’s proportional hazards model.

To compare attack, catch, and predation frequencies between the different species, contingency tables were constructed and analysed with Pearson chi-square test. For the leaf experiment each contact was considered an independent event. Results are presented using frequencies, which were calculated as follows: attack frequency was calculated as percentage of the number of contacts, and predation frequency was calculated as percentage of the number of attacks. For the tree experiment, the number of 3-hour observations in which two larvae were on the same leaf at least once was compared between treatments. In some 3-hour observations, the two larvae were observed on one leaf multiple times. These were treated as independent incidents in the subsequent statistical analysis for contact frequency because these occurred as separate incidents during the three hours. Contact frequency was calculated as the proportion of times a contact occurred when two individuals were present on one leaf; attack and predation frequencies were calculated similar to the leaf experiment. When the expected frequencies were too low to detect deviations from a discrete uniform distribution, Fisher’s exact test was used. All statistical analyses were performed with PASW Statistics (18.0.3, 9 Sept 2010). When multiple tests were performed on the same dataset, the critical p-value was Bonferroni-corrected.

## Results

### Leaf Experiment

The leaf experiment was performed 416 times. The total number of observations on each date varied between 6 and 66 (mean: 19). The number of replicates per treatment is given in table 2. In 23%–43% of the observations the larvae made contact (table 2). The time until contact differed significantly between combinations when all contacts were pooled (Log-Rank test, pooled pairwise comparisons, p = 0.008) (figure 2). However, when we distinguished between the first contact between two larvae and all later contacts between those two larvae, time until the first contact did not differ between combinations (Log-rank test, pooled pairwise comparisons, p = 0.088) while the time until a second or later contact did differ between combinations (Log-rank test, pooled pairwise comparisons, p = 0.007).

**Table 2:** Results of observations in leaf experiment.

	All observations				Contact observations			
Treatment	Number of observations		Contactfrequency	Total numberof contacts	Acting species	Attack	Catch	Predation
	without contact	with contact	(% ofobservations)			(% ofcontact)	(% ofattack)	(% ofcatch)
HH	37	28	43%	59	H	14%	0%	0%
CC	54	17	24%	33	C	15%	0%	0%
AA	39	20	34%	41	A	10%	0%	0%
HC	52	29	36%	38	H	24%	78%	86%
					C	5%	0%	0%
HA	42	24	36%	33	H	27%	100%	44%
					A	6%	0%	0%
CA	57	17	23%	23	C	9%	100%	100%
					A	0%	0%	0%

Attack, catch, and predation frequencies are presented for the observed contacts per treatment ( = species combination). Total number of replicates per treatment is the sum of the columns ‘without contact’ and ‘with contact’. During one observation, more than one contact can be made. Abbreviations: H = *H. axyridis*, C = *C. septempunctata*, and A = *A. bipunctata*.

**Figure 3:**
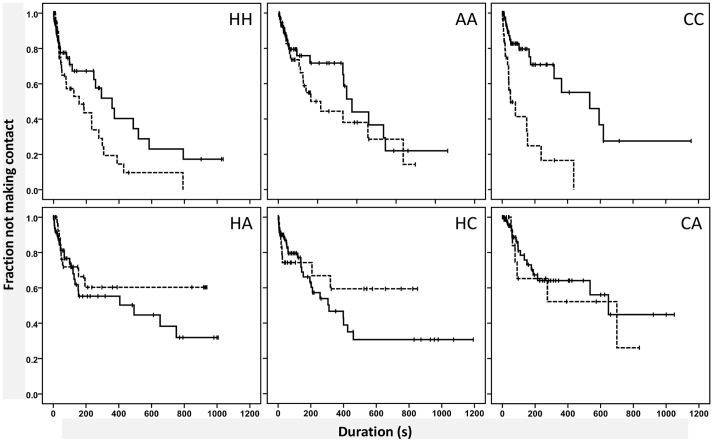
Effect of previous contact on time until contact. Survival curves of differences in time until first (solid line) and second or later contact (dashed line) are shown for each species combination. Two fourth-instar larvae were observed for 1000 seconds. Censored observations are marked with ‘+’. Abbreviations: H = *H. axyridis*, C = *C. septempunctata*, and A = *A. bipunctata*. Difference between first and second or later contact is significant (Log-rank test, pooled pairwise comparisons, p = 0.018).

**Figure 4:**
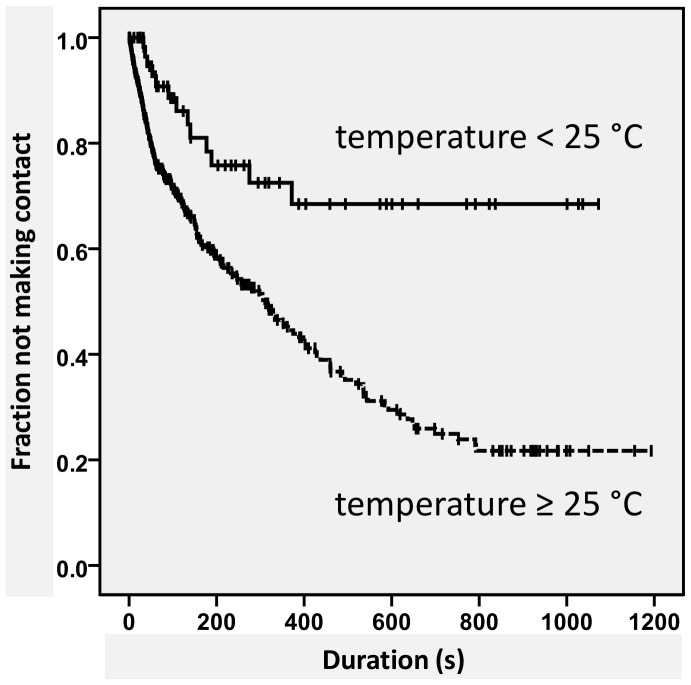
Effect of temperature on time until first contact. Survival curves of differences in time until first contact at temperatures below and above 25°C are shown for each species combination. Two fourth-instar larvae were observed for 1000 seconds. Censored observations are marked with ‘+’. Effect of temperature on time until first contact is significant (Cox’ regression model, Wald test = 13.521, df = 1, p<<0.001).

Further analysis per treatment showed that the time until subsequent contact differed from the time until first contact (Log-rank test, pooled pairwise comparisons, p = 0.018). Figure 3 shows that for the combinations of *H. axyridis* and heterospecifics, the time until the second or later contact was longer than the time until first contact, while for other combinations the time until subsequent contact was shorter than the time to initial contact. We also tested whether time to first contact was influenced by temperature. When temperature was high (≥25°C), the time until the first contact was 3.3 times shorter than when temperature was low (<25°C) (figure 4, Cox’ regression model, Wald test = 13.521, df = 1, p<<0.001).

When no contact was made, the observation could end in three ways: drop, leaving, or time-out. *Coccinella septempunctata* dropped earlier and more often than the other two species (Log-rank test, p<<0.001). The time until a larva left the leaf did not differ between species (Log-Rank test, pairwise comparisons, p>0.647) and only 11 observations ended in time-out. This number was too low to analyse the effect of species on time-out statistically.

### Aggressive Responses

**Figure 5:**
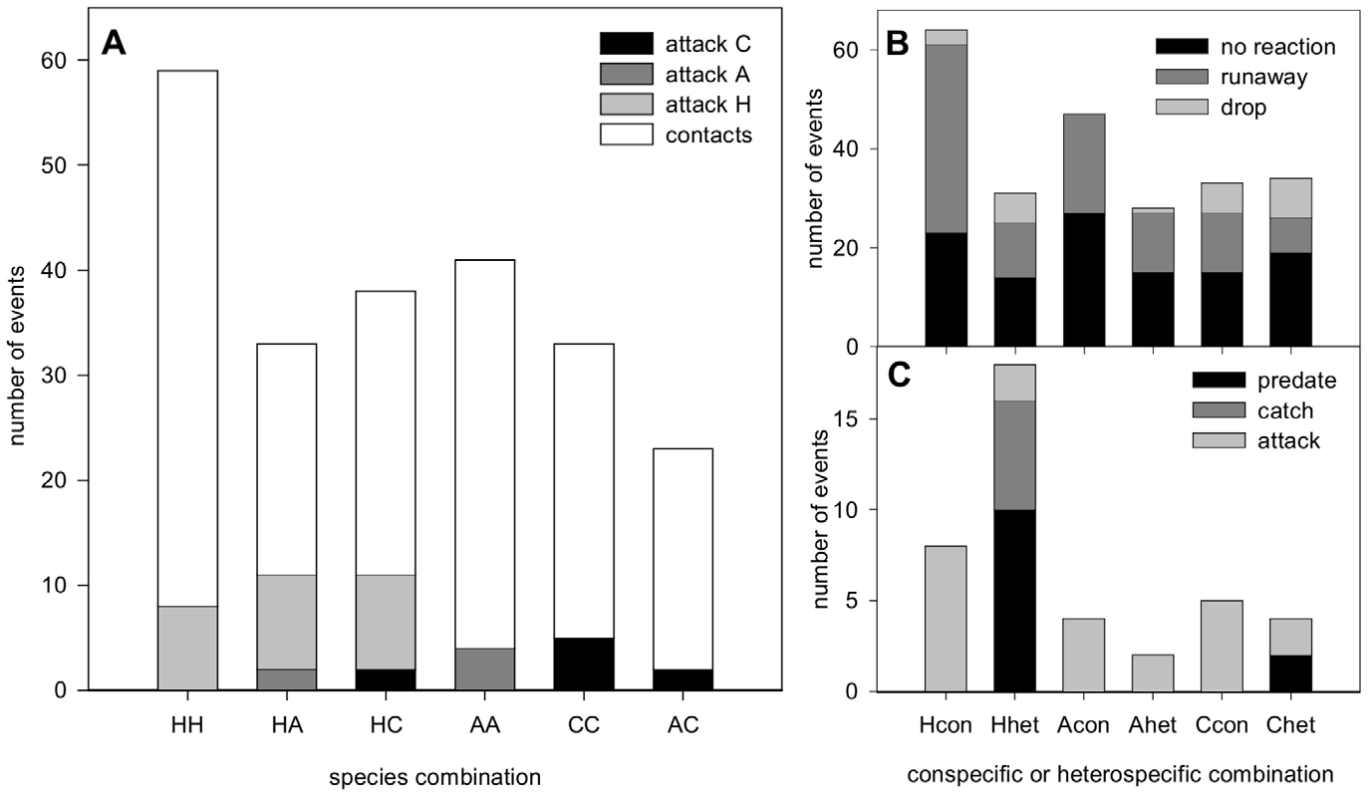
Behaviours observed in leaf experiments. Two fourth-instar larvae were observed for 1000 seconds on one leaf. A: Total number of contacts made per species combination during all observations. Total number of observations per species is given in table 2. B: Total number of non-aggressive responses after contact, when paired with conspecifics or heterospecifics (abbreviated to ‘con’ and ‘het’ respectively). C: Total number of aggressive responses after contact, when paired with conspecifics or heterospecifics (abbreviated to ‘con’ and ‘het’ respectively). Catch frequency of *H. axyridis* is significantly higher when paired with heterospecifics than when paired with conspecifics X^2^_1_ = 6.081, Fisher’s exact test p = 0.013). Abbreviations: H = *H. axyridis*, C = *C. septempunctata*, and A = *A. bipunctata*.

The number of contacts per treatment and the proportion leading to attack by either of the two larvae are shown in figure 5A. There was no difference in attack frequency between species, irrespective of the treatment (Χ^2^_2_ = 3.450, Pearson p = 0.178). However, when the species combination was taken into account, *H. axyridis* tended to attack heterospecifics more often than conspecifics (Χ^2^_1_ = 2.801, p = 0.094), while *Adalia bipunctata* and *C. septempunctata* attacked heterospecifics as often as conspecifics (Χ^2^_1_ = 1.560, Fisher’s exact test p = 0.205 and Χ^2^_1_ = 1.827, Fisher’s exact test p = 0.162 respectively, figure 5C). In addition, *H. axyridis* caught heterospecifics more often than it caught conspecifics (figure 5C, Χ ^2^_1_ = 6.081, Fisher’s exact test p = 0.013). This outcome could not be analysed for the other species due to the low number of catches. Both catches by *C. septempunctata* on heterospecifics (*A. bipunctata*) resulted in predation.

### Non-aggressive Responses

The number of non-aggressive responses after contact is shown in figure 5B. Pairwise Χ^2^-tests, not accounting for treatment, showed that *C. septempunctata* dropped after contact more often than *A. bipunctata* (Χ^2^_1_ = 12.677, Pearson p<0.001) and that *H. axyridis* ran away after contact more often than *C. septempunctata* (Χ^2^_1_ = 7.884, Pearson p = 0.005). The number of larvae showing no reaction after contact was similar for all three species (Χ^2^_2_ = 5.416, Pearson p = 0.067). The dropping frequency of all three species was not influenced by the treatment (Fisher’s exact test: Χ^2^_1_ = 0.567, p = 0.510; Χ^2^_1_ = 0.740, p = 1.000; and Χ^2^_1_ = 0.434, p = 0.552 for *H. axyridis, A. bipunctata,* and *C. septempunctata* respectively). In contrast, treatment did influence runaway frequency. For all three species the runaway frequency was higher in pairings with conspecifics than in pairings with heterospecifics (Pearson: p<0.001, p = 0.005, and p = 0.004 for *H. axyridis, A. bipunctata,* and *C. septempunctata* respectively). The frequency of larvae showing no reaction was only influenced by the treatment in case of *H. axyridis* and *A. bipunctata* and was higher in pairings with conspecifics (Pearson: p = 0.015 and p<0.001 for *H. axyridis* and *A. bipunctata*, respectively).

### Tree Experiment

**Figure 6:**
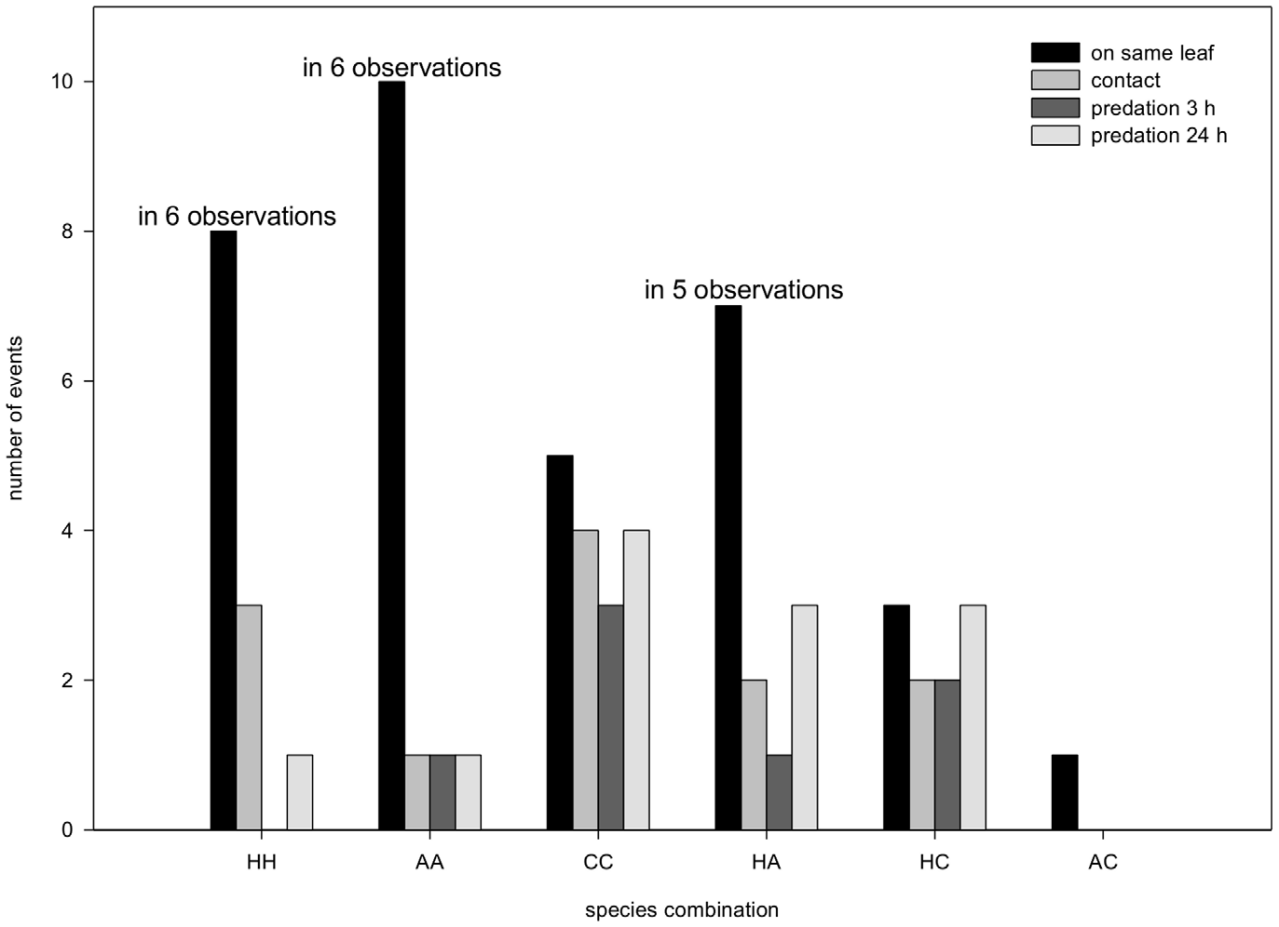
Incidents observed in tree experiment. Total number of incidents per species combination of two fourth-instar larvae observed on a lime tree during 540 time points (all data summed over 36 time intervals of 15 3-hour observations). Incidents are: ‘on same leaf’, ‘contact’, ‘predation after 3 hours’, and ‘predation after 24 hours’. The number of observations is indicated for the three treatments where the larvae were observed on the same leaf more than once. Abbreviations: H = *H. axyridis*, C = *C. septempunctata*, and A = *A. bipunctata*. Predation in the combination HA and HC was always by *H. axyridis.*

**Figure 7:**
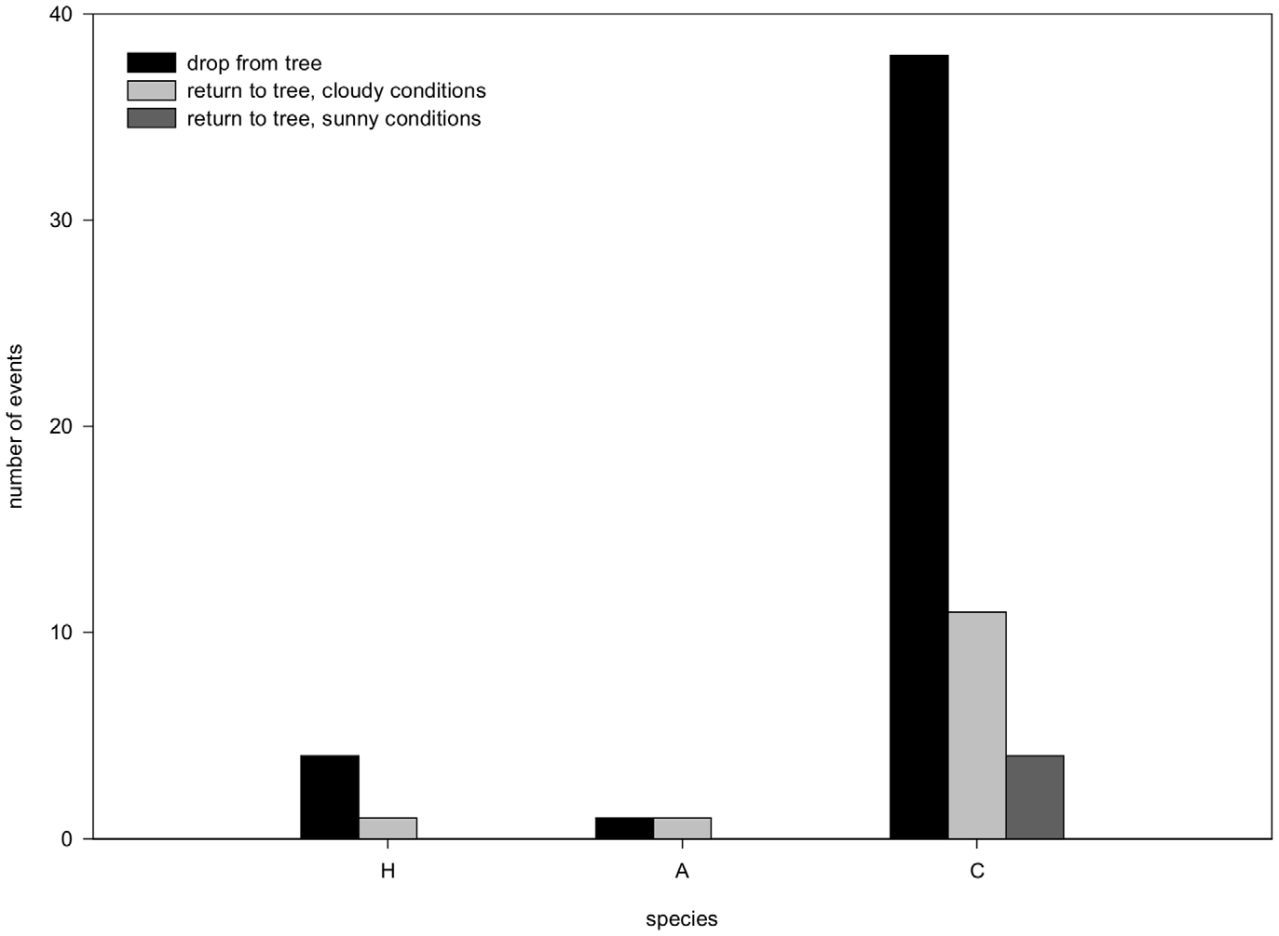
Larvae dropping from lime tree. Total number of incidents (dropping from and returning to lime tree) by fourth-instar larvae during 540 time points (all data summed over 36 time intervals during 15 3-hour observations per species combination). Abbreviations: H = *H. axyridis*, C = *C. septempunctata*, and A = *A. bipunctata*. Difference in drop frequency was significant between species X^2^_2_ = 89.902, Pearson p<0.001); difference in returning to tree between sunny or cloudy conditions was significant X^2^_1_ = 8.400, Pearson p = 0.004).

Location and behaviour was recorded at 540 time points (36 time intervals for 15 replicates) for each of the six treatments (N = 3,240 time points). Two larvae on one leaf were observed only one to ten times per treatment (figure 6), and this did not show a significant association with treatment (Χ^2^_5_ = 6.274, Fisher’s exact test p = 0.246). In seven out of 90 (15 replicates multiplied by six treatments) 3-hour observations two larvae were observed on one leaf more than once. Contact occurred 12 times in total; contact frequency (as percentage of being on same leaf) was not significantly associated with treatment (Χ^2^_5_ = 9.171, Fisher’s exact test p = 0.084). Seven of the 12 contacts resulted in predation, again this was independent of species combination (Χ^2^_4_ = 6.857, Fisher’s exact test p = 0.169). After 24 hours the number of predation incidents had almost doubled to 12. Differences in predation according to species could not be further analysed due to the low number of incidents, the large differences in dropping frequency between species and the low survival of larvae after 24 hours on the sand. Survival of *C. septempunctata*, in particular, was low (14 larvae out of 60 survived, with only 5 still on the tree). *Coccinella septempunctata* larvae dropped more often from the tree than larvae of the other two species (figure 7, Χ^2^_2_ = 89.902, Pearson p<0.001). Sometimes, the larvae (of all three species) were able to return to the tree. Interestingly, this happened more often when it was cloudy than when it was sunny (figure 3, tested for *C. septempunctata* Χ^2^_1_ = 8.400, Pearson p = 0.004).

## Discussion

### Contact between Larvae

Our results clearly show some remarkable differences from those of laboratory studies. The results of previous laboratory experiments showed higher contact frequencies than were observed in our study (e.g. [39]). Second, in laboratory experiments contacts between larvae result in behaviours that differ from those observed in the field due to limited escape possibilities. Generally, escape behaviours, such as fleeing, dropping from the plant, or retreating in refugia are important defensive mechanisms used by insects in the field [9], [12]. Therefore, it is difficult to predict field IGP-frequencies on the basis of laboratory results [25].

In most of our observations on individual leaves, one of the two larvae dropped or walked off the leaf before making contact (table 2, figure 4). Our observations suggest that if two larvae made contact, it was purely based on chance. No specific search pattern or type of prey recognition based on vision or olfaction was observed and, although larvae are able to perceive tracks of other larvae [34], they apparently only perceived the other larva when they touched them. Indeed, most studies report that ladybird larvae do not seem to perceive their prey before touching it ([40], [41], [42] and references therein, [43], [44]), or only react to alarm pheromone of their (crushed) prey over short distance [45], [46]. Visual information seems to be unimportant in prey-searching behaviour (pers. comm. J.L. Hemptinne), and olfactory cues have only been shown as contact pheromone between adult males and females [47]. However, the potential role of chemical communication between coccinellid larvae and their prey deserves more attention in future studies.

The likelihood of two larvae making contact was increased by the similar walking pattern of the three species. Our larvae showed a preference for walking along a vein or along the edge of the leaf, which is in line with the findings of earlier studies (e.g. [40], [43], [48]. In field conditions, this behaviour increases the chance of encountering aphids, as the density of lime aphids is higher near the veins [40].

The assumption of random contact is also supported by our observation that the time until first contact was similar for all species combinations. After first contact the two larvae are already near each other on the leaf, increasing the chance that they come into contact again, which explains why the time until second contact is generally shorter. Moreover, after the first contact between heterospecifics, *H. axyridis* predates regularly (4 out of 18 contacts with *A. bipunctata* and 7 out of 24 with *C. septempunctata*), while *H. axyridis* and *A. bipunctata* paired with conspecifics had second and later contacts in 50% of the cases. Predation excludes the possibility of a second contact and this explains the differences in time until second contact between treatments. During the 3-hour observations, all predation incidents were on the tree, and after 24 hours larvae that predated on the other larva were always found on the tree, while the larvae that did not survive were all found on the sand. This is a strong indication that scavenging on dead larvae did not happen during our experiment. The survival of larvae after 24 hours on the sand was low, in particular for *C. septempunctata* (only 14 larvae out of 60 survived, with 5 still on the tree). This low survival was probably caused by the dry and warm weather conditions.

In small arena experiments in the laboratory, contacts between two individuals occur often [39], while in most of the 90 observations of the tree experiment which more closely reflected field conditions, the two larvae were never observed on the same leaf. The larvae spent most time walking, in some cases up and down the whole tree. The (very) low number of observations of two larvae on the same leaf was probably caused by the 3-dimensional architecture of the habitat and by the size of the tree. The more complex habitat structure of trees − with branches, leaves, and possible refugia − is an important factor to consider when extrapolating results from laboratory to field [25].

### Response to Contact

Despite the low number of contacts observed in these experiments, when the outcome of all contacts is analysed, predation behaviour by *H. axyridis* was found to be quite high. *Harmonia axyridis* had more catches and successful predations than the other two species, and it mainly attacked and preyed upon heterospecifics (figure 2 and 7B). Overall, *H. axyridis* won in most encounters. The order of predation success is: *H. axyridis* > *C. septempunctata* > *A. bipunctata.* So, *H. axyridis* appears to be the most aggressive of the three species. The superiority of *H. axyridis* as an intraguild predator has also been reported in laboratory experiments with *A. bipunctata, C. septempunctata* (table 3) [20], [39], [49], and several other coccinellid species [10], [16], [50].

**Table 3:** Attack, catch, and predation frequencies in literature.

Combination	Acting species	Attack	Catch	Predation	Reference
		(% of contact)	(% of attack)	(% of catch)	
HH	H	40%	5%		Yasuda et al 2001
CC	C	5%	5%		Yasuda et al 2001
HC	H	55%	55%		Yasuda et al 2001
HC	C	15%	0%	0%	Yasuda et al 2001
HA	H	40%	87%	76%	Hautier 2003
HA	A	8%	85%	0%	Hautier 2003

Attack, catch, and predation frequencies are presented per species per species combination. Catch rates have been calculated using published escape rates. Abbreviations: H = *H. axyridis*, C = *C. septempunctata*, and A = *A. bipunctata*.

Several studies using molecular methods confirm that IGP occurs in the field ([51], [52], [53], Thomson et al. in prep.): *H. axyridis* collected at various sites contained 0%–53% exogenous alkaloids from *A. bipunctata, Adalia decempunctata* L. (Coleoptera: Coccinellidae), *Calvia spp., Coccinella septempunctata, Coleomegilla maculata* De Geer (Coleoptera: Coccinellidae), and *Propylea quatuordecimpunctata* L. (Coleoptera: Coccinellidae). Interestingly, *H. axyridis* also appeared as prey in *C. septempunctata, C. maculata,* and *P. quatuordecimpunctata*. These studies reported high levels of IGP, which is not surprising, as our observations were short compared with the duration of a full development cycle from egg to adult emergence. When considering the full lifespan, even a low contact and predation frequency could amount to an overall high number of contacts and predation incidents. Moreover, on our experimental tree with an average total branch length of 300 cm, a density of two larvae is low. During the aphid peak, wild larval densities may be ten to twenty times higher than in our experimental setup (pers. observation and pers. comm. P.W. de Jong). In addition, model simulations have shown that even very low frequencies of encounters and predation events may lead to a large differences in fitness in favour of the intraguild predator. Thus, IGP might play a substantial role in the invasion success of *H. axyridis* [54]. With longer durations of possible contact the total probability of contact occurring will increase. After 24 hours the number of predation incidents had increased from 7 (after 3 hours) to 12. So, despite the low number of contacts observed, predation was recorded in 12 out of 90 observations.

The multiple defence lines of *H. axyridis* (size [10]; chemical deterrence [32], and morphological structure (spines) [24]) combined with its aggressive attack behaviour might explain the higher IGP-frequencies of *H. axyridis* against heterospecifics as compared to conspecifics. As *H. axyridis* established in Europe recently, defences of native ladybird species against predation by *H. axyridis* have not yet co-evolved [10].

Results from earlier laboratory experiments and from our semi-field experiments are in line with knowledge of the defence mechanisms used by ladybirds: *A. bipunctata* is the weakest species since its chemical defence is not effective when paired with *H. axyridis* [22], and *C. septempunctata* can protect itself reasonably well as its general dropping behaviour seems to be effective when paired with *H. axyridis*. Remarkably, after being caught by *H. axyridis, A. bipunctata* managed to escape from a catch by *H. axyridis* in five out of nine incidents in our study, whereas *C. septempunctata* managed to escape after being caught by *H. axyridis* only once in seven incidents. Yasuda [20] also reports lower escape rates of *C. septempunctata* larvae from attacks by *H. axyridis.* Fourth instar larvae of *A. bipunctata* are equally able to escape an attack by *H. axyridis* as *H. axyridis* is able to escape an attack by *A. bipunctata,* whereas younger instars are less successful in escaping [49].

Species combination did not influence dropping frequency after contact. The other two non-aggressive responses (runaway and no reaction), occurred more often in pairings with conspecifics than with heterospecifics. This lower frequency of non-aggressive responses in heterospecific pairings might indicate that for all species − not only for *H. axyridis* as we have shown − the frequency of aggressive responses is higher in heterospecific pairings, but due to low numbers of observations we could not statistically test for differences.

### Dropping Behaviour

In both experiments, larvae of *C. septempunctata* often dropped from the leaf, corroborating results of Sato et al. [22]. When dropping occurred after contact, we considered it escape behaviour, but some individuals also fell without any apparent cause. Sato et al. [14] observed that *C. septempunctata* emigrates from the plant sooner than other species when aphid density decreases, thus reducing the occurrence of IGP and cannibalism. It is not clear whether this emigration was caused by larvae dropping from the plant or walking off the plant. *Harmonia axyridis* and *A. bipunctata* prefer trees and shrubs [22], [42]. For the arboreal species *A. bipunctata* the risk of falling is low as the larvae have a large anal disc for holding onto leaves [55], [56], [57]. We observed that *H. axyridis* larvae were also capable of holding onto the leaf with their anal disc, as has been suggested by Osawa [58]. *Coccinella septempunctata* prefers herbaceous vegetation over trees as host plant [41], [42] which may explain their frequent dropping behaviour: it is easy to return to the host plant when vegetation grows close to the ground. Further, *C. septempunctata* is able to forage for aphids on the ground, and this ground foraging is estimated to provide them with 30% of the daily diet in wheat [59].

The results of this study show that *H. axyridis* wins in most encounters with heterospecifics and is the strongest intraguild predator of the species tested here. Being an invasive species in Europe and North America, the strong IGP-pressure of *H. axyridis* potentially affects the balance between this invasive predator and native intraguild predators within the aphidophagous guild [cf. 60]. This may result in reduced diversity of native coccinellids in this guild, but does not have to result in changes in aphid densities. There are reports of improved control of pest aphids after release and establishment of *H. axyridis*, but whether non-damaging aphid densities are more likely to increase or decrease following changes in coccinellid diversity is not yet clear [61]. Most IGP studies, including those conducted at (semi-) field level, are conducted on the level of individual interactions. Although studies in semi-field conditions give more realistic results than laboratory-based experiments, extrapolation to community and ecosystem level is needed to fully understand the effects of an invasive species on aphidophagous guild and its ecosystem service of aphid suppression. Large field community experiments, in combination with modelling studies such as those described by [60], [62] might provide this understanding.
